# Mr. Bingham's Practical Essays on Strictures of the Urethra, Diseases of the Testicles, &c. &c.

**Published:** 1820-12-01

**Authors:** 


*Practical Essays on Strictures of the Urethra and Diseases of the Testicles, including, Observations on Fistula in Perinœo and Hydrocele. Illustrated by numerous Cases and an Engraving; and prefaced with some Remarks on Life and Organization.* By Robert Bingham, Fellow of the Royal College of Surgeons, London. Octavo pp. 357. 1820.

The misery and fatality produced by diseases in the generative organs, invite us to examine every attempt designed to extend our knowledge and improve our practice in this department. Some of those which are peculiar to our own sex, are believed by the multitude, to be invariably the offsprings of vice, and the just punishment of moral depravity. Hence we find, throughout society, that less sympathy is extended towards these unfortunate sufferers, than any others. The disciples of Epicurus, however, are not the exclusive victims; and, to compensate for the disadvantages of vulgar prejudice, these several afflictions have received the most profound attention from the wisest surgeons the world ever produced, namely, Hunter and Abernethy. Intellectual excellence, being the gift of Heaven, sheds its splendour on every object within the sphere of its influence: as the sun, which shines alike on the poor and the rich, the fool and the philosopher.

At first sight there does not appear to be much connexion between strictures in the urethra and remarks on life and organization; but, as Mr. Bingham wishes to establish a certain theory, he has thought proper to introduce the latter subject, that he may unfold his views the more readily respecting the cause of structural derangements. He briefly, but ably, supports the doctrine of anti-materialism, and then observes,

"The mere life of every part depends entirely upon a constant supply of fresh blood, but the function of every part depends chiefly upon the supply of nervous influence. Hence we must conclude, that life produces all its partial or peculiar effects through the medium of the nerves, which induces a further conclusion, that all local disease commences in morbid, nervous action." *Preface*, p. xiii.

Etiology. Three varieties of stricture are noticed by Mr. Bingham; the spasmodic, the permanent, and the mixed. Dissection enables us to discover other distinctions, which have been so often described, that we shall not stop to explain them here, being desirous of hastening to the author's theory of the disease. From the following passage, we conclude that he is not a convert to Mr. Charles Bell's doctrine of the non-muscularity of the urethra.

"It is well known, that nervous irritation in muscular parts, often occasions partial and obstinate contractions; and when this effect takes place in the urethra, I believe it constitutes, what is generally understood by the term, spasmodic stricture." P. 7.

He is disposed to think, that *nervous irritation* must always exist first, and that a permanent, is almost always preceded by a spasmodic, stricture. Some of what are called permanent strictures are, in his opinion, not occasioned by any alteration of structure, but by a closer and more compact texture of the fibres. In other instances, complete structural derangements are found resembling cartilage, which he supposes must be the result of a *peculiar, but inexplicable, morbid nervous action.*

The belief that stricture *always* proceeds from a venereal disease, is now very justly exploded by all well-informed surgeons. Irritating and astringent injections, we fear, are more frequently the cause of stricture, than is generally understood. We have lately been consulted by a gentleman, who laboured under most distressing *nervous irritation*, as Mr. Bingham calls it, in the urethra, testicles, and rectum, produced by one injection of green tea-infusion, and terminating in spasmodic stricture.

The passing of urinary calculi along the ureters or urethra, will sometimes occasion spasmodic stricture in the latter part; and we have a patient, who always knows that he has crystals of uric acid in his bladder, by a temporary stricture in the urethra. The earthy phosphates too, in our own practice, have always been accompanied by symptoms resembling stricture.

An abuse of the functions of the urethra appears to be the most common of all the causes of stricture, particularly in hot climates, where venereal excesses are common.

Disorders of the digestive organs, prostate gland, bladder, kidneys, &c. are also found to be frequent causes of stricture.

Symptomatology. A contracted stream of urine is always attendant, and irritability of the bladder frequently to so great a degree, that the urine is discharged every hour, or oftener. The latter symptom, however, is common in old men, from a chronic inflammation of the prostate gland, and is aggravated by riding on horseback, by piles, and constipated bowels.

As in stricture of the rectum, so in that of the urethra, we often meet with a mucous discharge, which we suppose proceeds from a chronic inflammation in the mucous membrane.

Various sensations are produced in different degrees by stricture in the urethra; and sympathetic affections in contiguous and distant parts are connected with it. The disorders in the adjoining parts are well understood; those in distant organs have been less alluded to. The doctrine of the sympathies of the different organs may be said to have originated with Mr. Hunter; and it would be superfluous for us, if it were possible, to explain the benefit Mr. Abernethy has conferred on our art by his minute attention to the diseases connected with a disordered condition of the chylifactive viscera. This nosopoietic influence of gastric derangements is perhaps more universally admitted than understood.

The symptoms of stricture being uncertain, our enumeration of them has been slight. When the disease is suspected, the only decisive proof we can have of its presence is to be obtained by a careful examination of the urethra by a bougie. For this purpose Mr. Bingham says, a large soft bougie should always be employed, which will receive the impression of the stricture, and point out its nature, extent, and situation. The soft bougie is prepared by immersing a common plaster-bougie a few minutes in warm water.

Treatment. To remove diseased actions, and to restore the morbid parts to their original state, are the grand objects to be had in view in the cure of stricture. Every thing found to aggravate the disease must be avoided, and particular attention must be paid to the stomach and bowels, the disorders of which will be found to affect the urethra both by contiguous and remote sympathy. With the view of preserving the parts in a state of quiescence, Mr. B. recommends

"The minds of strictured patients to be kept as free as possible from venereal ideas." P. 44.

We fear Mr. B. will find this a difficult matter, for stricture is usually met with at a period of life, when the passions are strong, and reason imperfectly matured.

The local means of cure may be divided into mechanical and chemical. Of the first kind are bougies, which are of four different species: the plaster, catgut, flexible gum, and metallic. We think with Mr. B. that in general they should be made conical for about an inch or an inch and a half, and then be continued of the same diameter to the other end. Catgut-bougies we conceive are dangerous instruments, when used for the purpose of *dilating* a stricture; indeed a forcible dilatation ought never to be attempted. With respect to the metallic bougies, it must be observed that they are liable to break from frequent bending and other causes; and this accident happened to a metallic catheter in a patient of Mr. J. M. Coley's, at Bridgnorth, on whom he had operated for fistula, in perinæo. The fractured part had been exposed as long as five or six weeks to an ulcerated surface, and seemed to be decomposed, acquiring a brittleness and scaly appearance. The portion which lay in the bladder remained sound. It broke off near the bulb of the urethra, and he extracted it by means of a piece of strong brass wire, in the shape of a catheter, and introduced with the convex side towards the pubes. Mr. Coley recommends the catheter to be renewed every third week.

On some occasions Mr. Bingham employs a bougie, which, he says, possesses some good qualities peculiar to itself.

"This may be termed the hollow or compound bougie, as it differs in no respect from the flexible gum catheter, but in having no eye or aperture near its point. The various kinds of stilets which we may employ, give us great power of regulating the flexibility and strength of this instrument; and by withdrawing the stilet a little way, we shall obtain a flexible point of as much firmness as is desired." P. 59.

To facilitate the introduction of bougies, Mr. B. makes use of ceratum cetacei. They should also be warmed and softened, and the point bent a little upwards; but very small ones should be used cold, when it is necessary that they should retain any peculiar curvature. Large ones require to be bent nearly in the shape of a catheter, before they can be safely employed. When the point arrives at the stricture, we must make the most gentle attempts to convey it through the obstruction, removing from our minds the idea of *dilatation*, a term which, in our opinion, ought to be exploded from the young surgeon's nomenclature. If the bougie is too large, we must be satisfied with pressing its point against the orifice of the contraction; and, having waited a day or two, we may renew our attempts with a smaller instrument. On the approach of a bougie, every stricture takes on a spasmodic action, which often communicates to the urethra in front a disposition to contract. By withdrawing the bougie a little, a tendency to dilate is excited, and during this spontaneous dilatation, if the point be carefully propelled again, it will generally enter the obstruction. We have been thus minute, in order to impress on the inexperienced that the great secret in the use of bougies consists in stealing through the stricture by art and management, and not by endeavours to force a passage. By the conduct we have recommended and long practised, we are convinced that no accident can happen either to the urethra or bougie.

An expedient has occurred to Mr. B. similar to that adopted by Mr. Coley, and described in our last No. under the head, White on Stricture in the Rectum. Mr. B. thinks the canula, to convey the bougie down to the stricture, should be flexible; but he does not appear to have had recourse to it. When the contraction requires a very slender bougie, and the latter cannot be introduced without difficulty, we should certainly try the canula, which would defend the bougie from heat and obstructions, and conduct its point up to the very orifice of the stricture.

When the argenti nitras, one of the chemical means of cure, is made use of, Mr. B. advises it to be powdered and applied on the point of the bougie, which must be previously warmed. To guard the sides of the urethra from the caustic, the bougie is to be concealed within an elastic gum-canula. By the same apparatus, an ointment, composed of argenti nitr. from two to eight grains, and adeps, half a drachm, may be brought into contact with the whole of the strictured surface. If the caustic has not been applied, care should be taken not to suffer the quantity of it to exceed at first two grains. Where the flexible canula cannot be had, a grooved bougie, the ointment being deposited in the groove, will form a substitute.

Mr. Bingham presents us with an account of the manner of preparing and using the bougie armed with kali purum; and concludes with sensible remarks on the modus operandi of the remedy, in which we coincide. He has not found it produce any better effects than the potassæ subcarbonas; which may be applied by means of the elastic gum-canula, like the argenti nitras. By some mistake the term *potassa* subcarbonas occurs constantly in Mr. Bingham's book instead of *potassæ* subcarbonas. Three cases are introduced to exhibit the good effects of this application. Sodæ subcarbonas exsiccat. and sodæ carbonas have also been tried in stricture; and their operation has been similar to that of the subcarbonate of potash. Three cases, in which the sodæ subcarb. exsic. was used, are added.

Another auxiliary to the bougie, which Mr. B. believes has never before been employed for the cure of stricture, is strongly recommended by him. It is the ung. hydrarg. fort.

" The manner of applying the unguentum hydrargyri fortius is to smear it upon the bougie, and to pass it through the stricture, where it may be allowed to remain for a longer or shorter period, according as it is wished to exert more or less influence.

" Sometimes it produces no sensation different from that which the bougie alone would excite: at other times, patients have told me it felt warm, and sometimes very warm, but never amounting to painful sensation." P. 179.

Mr. B. was first led to try this remedy from observing its infallibly good effect when applied to phymosis, which he believes to be of the same nature with some urethral strictures. He thinks it merits the preference before all other expedients in strictures impenetrable to a bougie, although open for the discharge of the urine.

Cold or tepid hip-baths may be useful, if properly adapted to the age and constitution of the patient.

In the observations on *fistula in perinæo* and on *false passages* in the urethra, nothing new or particularly interesting occurs. The species of the former disease described by Mr. B. appear to be of a comparatively mild and chronic nature. There is a most formidable kind of disease, which we have frequently met with, occurring in the perinæum. It begins with a small, hard lump in the urethra, which is either painful or smarts after every discharge of urine. In a few days it acquires a larger size, and feels as hard as a stone. By degree it becomes a little diffused: the urine has escaped into the corpus spongiosum urethræ. At the end of a week an erysipelatous tumefaction spreads about the scrotum and penis; irritative fever of the worst kind commences; and vesications and mortification follow. If the experienced surgeon is called in at this crisis, he cuts through the smaller integuments down to the abscess; he plunges his knife deep a second time, and matter and urine burst forth with an intolerable fœtor. The catheter is now to be introduced twice or three times a day, and, the sloughs having separated, the wound is to be healed by proper dressings. Towards the last, the hydr. oxymur: in the form of lotion is an excellent application to stimulate the fistulous aperture. When severe and frequent *rigors* occur after the sloughing process is complete, we suspect matter is forming in the prostate gland; and if the pulse is at 130, and delirium and carphologia are present, the case will terminate unfavourably. These rigors, which are often followed by a hot and perspiring state, are to be distinguished from idiopathic fevers by the frequency and irregularity of their occurrenc

*Hernia humoralis* produced by sympathy with stricture is often speedily removed by the introduction of the bougie. We suspect that few strictures are found unconnected with some affection of the testis. Most of our own patients have had a small hard tumour on the epididymis, and occasional tumefaction in the spermatic chord. These symptoms, together with pain in both those situations often continue many years, before the cause of them is discovered. The bougie removes both the stricture and its consequences.

Mr. B. informs us that Mr. Ridgway cures his patients, who have gonorrhœa and hernia humoralis, with an injection of *argenti nitras*, which is prepared by dissolving from ten grains to forty in an ounce of distilled water. It must be used with an ivory syringe, and not thrown further than four inches,'nor repeated oftener than twice a day.

Mr. Bingham's book closes with some remarks on *suspensory bandages*, and on *hydrocele*. As an improvement in the former, he recommends soft leather in preference to linen or callico. In speaking of hydroceles, he says, he is persuaded that there are some cases, in which an operation is unnecessary. We think so too, and in our own practice we have been able to cure every case of infantile hydrocele by the daily application of linim. hydrarg. comp. This plan, however, does not succeed with adolescents or adults.

Mr. Bingham's book will be found useful to the young and inexperienced, and interesting to those, who make the cure of strictures their principal study. The practice he recommends is safe, his speculations are ingenious, and on the whole his work displays considerable talent. Before he publishes his essay on the prostate gland, from which we expect much information, we advise him to reduce it into the smallest compass, consistent with perspicuity. His language is good, but his style is rather diffuse; and every one knows that what is gained by expansion is lost in force.

" Quidquid præcipies, esto brevis: ut cito dicta
" Percipient animi dociles, teneantq. fideles."

**2. *Horatii Flacci de Arte Poet. Epis.***

The style of Celsus is well worthy the study of those who wish to obtain credit, or to establish facts by medical writings. The great art in pleasing our fastidious palates in this Augustan age consists in supplying us with such literary food, as will not alarm us by its quantity, nor by its flatulent quality produce indigestion or the vapours.

